# A Hybrid Wavelet Transform Based Short-Term Wind Speed Forecasting Approach

**DOI:** 10.1155/2014/914127

**Published:** 2014-07-21

**Authors:** Jujie Wang

**Affiliations:** School of Economics and Management, Nanjing University of Information Science and Technology, Nanjing, Jiangsu 210044, China

## Abstract

It is important to improve the accuracy of wind speed forecasting for wind parks management and wind power utilization. In this paper, a novel hybrid approach known as WTT-TNN is proposed for wind speed forecasting. In the first step of the approach, a wavelet transform technique (WTT) is used to decompose wind speed into an approximate scale and several detailed scales. In the second step, a two-hidden-layer neural network (TNN) is used to predict both approximated scale and detailed scales, respectively. In order to find the optimal network architecture, the partial autocorrelation function is adopted to determine the number of neurons in the input layer, and an experimental simulation is made to determine the number of neurons within each hidden layer in the modeling process of TNN. Afterwards, the final prediction value can be obtained by the sum of these prediction results. In this study, a WTT is employed to extract these different patterns of the wind speed and make it easier for forecasting. To evaluate the performance of the proposed approach, it is applied to forecast Hexi Corridor of China's wind speed. Simulation results in four different cases show that the proposed method increases wind speed forecasting accuracy.

## 1. Introduction

Special attention has been focused on renewable energy due to environmental deterioration and conventional resource depletion. Wind power is a clean and nonpolluting renewable energy source. Recently, the amount of energy generated by wind power has rapidly increased. The installed wind power capacity increased by nearly 200% between 2005 and 2009 [1, 2]. It is expected that about 12% of the total world electricity demands are to be supplied from wind energy resources by 2020 [3]. However, operation of wind power generation is very challenging because of the intermittent and intrinsic complexity nature of the wind speed [4]. Fluctuating wind speeds make it difficult to predict how much power will be injected into a distribution network, which can result in energy transportation issues [2, 5]. This problem can be significantly mitigated if the operation of wind farm can be controlled based on the accurate information of dynamic wind speed forecasting [6]. In addition, integration of wind power into an electrical grid requires an estimate of the expected power from the wind farms at least one to two days in advance [7]. Short-term wind speed forecasting is an extremely important field of research for the energy sector. As a result, it is becoming increasingly important to obtain accurate short-term wind speed forecasting.

In order to improve forecasting accuracy of wind speed, many approaches have been developed in the past 30 years. Generally, these approaches can be divided into two categories: statistical methods and artificial intelligence (AI) methods. The statistical methods, mainly including persistence method (PM) and autoregressive integrated moving average models, are used for wind speed forecasting using statistical equations to describe the statistical regularities of wind speed [8–11]. These approaches have some advantages such as simplicity and being easy to model and they do not require any data beyond historical wind speed data [8, 12–17]. However, the forecasting accuracy of these approaches drops fast when the nonlinear characteristics of wind speed series are obvious. To overcome this limitation of statistical approaches, the artificial intelligence (AI) techniques, mainly including artificial neural network (ANN), have attracted more attention for wind speed forecasting and have also been determined to be more accurate as compared to statistical models [5, 18–27]. Unlike statistical models, ANN is data-driven and nonparametric model. It does not require strong model assumptions and can map any nonlinear function without a priori assumption about the properties of the data [28–30]. Furthermore, Chester [31] proved that two-hidden-layer neural network (TNN) appears to provide higher accuracy, better generalization, and fewer total processing nodes than a single-hidden-layer network. These results encourage us to use TNN for our studies of wind speed forecasting.

When using some models for wind speed forecasting, the observed original values of forecasting variables are usually directly used for building forecasting models. However, due to the fluctuation and complexity of wind speed, it is difficult to capture its nonstationary property and accurately describe its moving tendency. To improve the forecasting precision, the multiscale decomposition of original wind speed is indispensable. A wavelet transform technique (WTT) is a relatively new field in signal processing [32]. The WTT decomposes a signal into different scales, making it useful in distinguishing seasonality, revealing structural breaks and volatility clusters, and identifying local and global dynamic properties of a signal at specific timescales [33]. The WTT has been shown to be an essential tool for data preprocessing and has been widely used in extracting the basic characteristics from the nonstationary time series [34]. For this reason, this study applies a WTT to decompose the wind speed time series.

In this paper, a hybrid model known as WTT-TNN is proposed for wind speed forecasting. In the first step of the approach, a WTT is used to decompose wind speed into an approximate scale associated with low frequency and several detailed scales associated with high frequencies. The approximated scale reveals the trend, while the detailed scales tend to be related to seasonal influences and exogenous variables effect. In the second step, a TNN is used to predict both approximated scale and detailed scales, respectively. In order to find the optimal network architecture, the partial autocorrelation function (PACF) is adopted to determine the number of neurons in the input layer, and an experimental simulation is made to determine the number of neurons within each hidden layer in the modeling process of TNN. Afterwards, the final prediction value can be obtained by the sum of these prediction results. In this study, a WTT is employed to extract these different patterns of the wind speed and make it easier for forecasting. To evaluate the performance of the proposed approach, it is applied to forecast Hexi Corridor of China's wind speed. Compared with the persistence method (PM), the one-hidden-layer neural network (ONN), and the TNN, simulation results in four different cases show that the proposed method increases wind speed forecasting accuracy.

The rest of this paper is organized as follows.  Section 2 presents the WTT-TNN approach for wind speed forecasting.  Section 3 provides the evaluation criteria which were used to evaluate the prediction accuracy.  Section 4 presents the numerical results from four real datasets. Finally, Section 5 outlines the conclusions.

## 2. Proposed Approach

**Figure 1:**
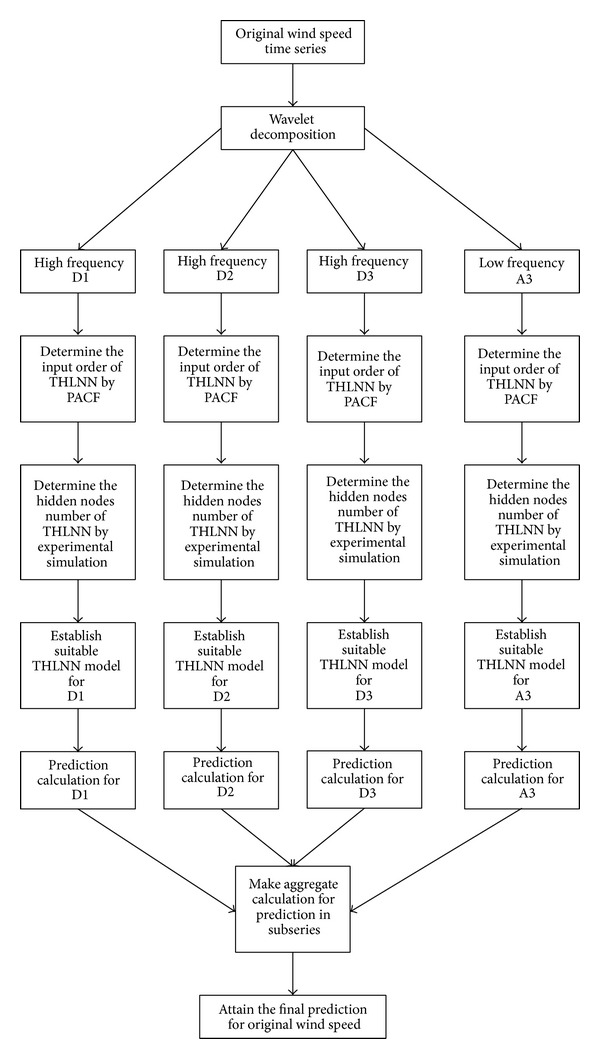
The flowchart of the proposed approach.

In this paper, the WTT-TNN approach, which applies the WTT to TNN, is proposed for short-term wind speed forecasting. The algorithm is described as follows and the flowchart is shown in Figure 1. The methods used in the WTT-TNN approach are briefly introduced in the following subsections.

*Step 1*. Apply the WTT to decompose an original time series into a set of different subseries which can be identified, separately predicted, and recombined to get aggregate forecasting. For example, three decomposition levels are shown in Figure 1. From Figure 1, it can be seen that an original wind speed time series has been decomposed into a low-pass filter (A3) and three high-pass filters (D1, D2, and D3).

*Step* *2*. Use the TNN to build a forecasting model for each subseries and make the prediction in each subseries. To determine the input order of TNN, the PACF is adopted for each subseries. On the other hand, to determine the hidden nodes number of TNN, an experimental simulation is made with different kinds of nodes combination for each subseries.

*Step 3*. Conduct aggregate calculation for the forecasting results in the subseries to attain the final forecasting for the original time series.

*Step 4*. Compare the performance of the WTT-TNN model with a PM, ONN, and TNN.

### 2.1. Wavelet Transform Technique (WTT)

A WTT is an essential tool for data preprocessing and has been widely used in the fields of image processing, signal processing, and time series analysis [35–40]. The WTT allows the decomposition of a signal into different levels of resolution scales, which means that we can extract the required data components. To be specific, the WTT converts a wind speed series into a set of constitutive series. These constitutive series present a better behavior than the original wind speed series, and therefore they can be predicted more accurately. The reason for the better behavior of the constitutive series is the filtering effect of the WTT. In this section, a brief summary of WTT is presented.

As a special kind of Fourier transform, WTT has been successfully applied to decompose the signals in different scales. The WTT has two kinds; one kind is continuous wavelet transform (CWT) and the other is discrete wavelet transform (DWT). The definition of the CWT is described as follows [41]:

(1) $$\begin{array}{c} \text{CW}{\text{T}}_{x}^{\psi }\left(b,a\right)=\varphi_{x}^{\psi }\left(b,a\right)=\frac{1}{\sqrt{\left|a\right|}}\int x\left(t\right)\cdot \psi^{∙}\left(\frac{t-b}{a}\right)dt, \end{array}$$

where *a* and *b* are the scale parameter and the translational parameter, respectively, and ∙ is the complex conjugate of *ψ*(*t*). If *a* = 1/2^*s*^ and *b* = *k*/2^*s*^, then a discrete version of (1) is denoted as follows:

(2) $$\begin{array}{c} \text{DW}{\text{T}}_{x}^{\psi }\left(k,s\right)=\varphi_{x}^{\psi }\left(\frac{k}{2^{s}},\frac{1}{2^{s}}\right)=\overset{\infty }{\underset{\infty }{\int }}x\left(t\right)\cdot \psi^{∙}\left(\frac{t-k/2^{s}}{1/2^{s}}\right)dt, \end{array}$$

where *s* ∈ *Z* and *k* ∈ *Z* (*Z* denotes the integer set). The DWT can meet the multiresolution decomposition at various scales and can decompose the signal in different parts. In this study, the DWT can decompose the wind speed series in several scales, where both the approximated and detailed parts of the data are obtained. The approximated scale reveals the trend, while the detailed scales tend to be related to seasonal influences and exogenous variables effect. Afterwards, the TNN model can be adopted for forecasting in the approximated scale and the detailed scales, respectively.

### 2.2. Two-Hidden-Layer Neural Network (TNN)

**Figure 2:**
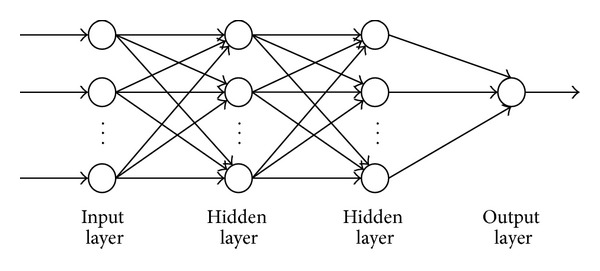
The architecture of the TNN.

A TNN generally consists of four layers, an input layer, two hidden layers, and an output layer. Each of those layers contains nodes, and these nodes are connected to nodes at adjacent layer(s). The basic architecture of a TNN is shown in Figure 2. The calculated process can be described as follows.

Assume that there are *n* input neurons in the input layer, *m*_1_ hidden neurons in the first hidden layer, *m*_2_ hidden neurons in the second hidden layer, and one output neuron in the output layer; a calculation process can be described by two stages [42].

*(I) Hidden-Layer Stage*. The outputs of all neurons in the second hidden layer are calculated by the following steps:

(3) $$\begin{array}{c} g_{1j}=f_{H1}\left(\overset{n}{\underset{i=0}{\sum }}u_{ij}x_{i}\right)\;j=1,2,\ldots ,m_{1},\\ g_{2k}=f_{H2}\left(\overset{m_{1}}{\underset{j=0}{\sum }}v_{jk}g_{1j}\right)\;k=1,2,\ldots ,m_{2}, \end{array}$$

where *x* = [*x*_1_, *x*_2_,…, *x*_*n*_] is the input value in the input layer, *g*_1*j*_ is the output value of the* j*th node in the first hidden layer, *g*_2*k*_ is the output value of the* k*th node in the second hidden layer, *u*_*ij*_ is the weight value between the *i*th node in the input layer and the* j*th node in the first hidden layer, *v*_*jk*_ is the weight value between the* j*th node in the first hidden layer and the* k*th node in the second hidden layer, and *f*_*H*1_ and *f*_*H*2_ are the activation functions in the two hidden layers. In general, *f*_*H*1_ is the hyperbolic tangent transfer function in the first hidden layer, and *f*_*H*2_ is the logarithmic sigmoid transfer function in the second hidden layer.

*(II) Output Stage*. The output of the output layer is given as follows:

(4) $$\begin{array}{c} y=f\left(\overset{m_{2}}{\underset{k=0}{\sum }}\omega_{k}g_{2k}\right), \end{array}$$

where *ω*_*k*_ is the weight value between the* k*th node in the second hidden layer and the output layer, *y* is the output value of the output layer, and *f* is the activation function, usually a linear function.

Backpropagation is a common method of training ANN [42, 43]. The learning algorithm considered herein is the backpropagation. In this study, all the data have been normalized, and all weights are assigned to random values initially and then modified by the delta rule according to the learning samples. In order to find the optimal network architecture, the PACF is adopted to determine the number of neurons in the input layer and an experimental simulation is made to determine the number of neurons within each hidden layer. For more detailed information about TNN model, please refer to [44, 45].

### 2.3. Partial Autocorrelation Function (PACF)

In ANN theory, apart from the structure of network, the training data format also can affect the performance of network directly. Once the calculation of the WTT is finished, several subseries can be attained. How to use those subseries data to train a neural network is another important work. In order to overcome the limitation of ignoring the relationship between input(s) and output(s) of ANN, inspired from the identification of parameter *p* in ARMA (*p*, *q*) model (see (5)), a PACF is utilized to identify the inputting data structure of the ANN models [46]. Concretely, assuming that *x*_*i*_ is the output variable, if the partial autocorrelation at lag *k* is out of the 95% confidence interval which is $\left[-1.96/\sqrt{N},1.96/\sqrt{N}\right]$ approximately, *x*_*i*−*k*_ is one of the input variables. The description of PACF is as follows [47, 48]:

(5) $$\begin{array}{c} \varphi \left(B\right)x_{t}=\theta \left(B\right)a_{t}, \end{array}$$

where

(6) φ(B)=1−φ1B−φ2B2−⋯−φpBp,θ(B)=1−θ1B−θ2B2−⋯−θqBq.

For a time series {*w*_1_, *w*_2_,…, *w*_*n*_}, the covariance at lag *k* (if *k* = 0, it is the variance), denoted by *γ*_*k*_, is estimated in

(7) $$\begin{array}{c} {\overset{\wedge }{\gamma }}_{k}=\frac{1}{n}\overset{n-k}{\underset{i=1}{\sum }}\left(w_{i}-\overset{-}{w}\right)\left(w_{i+k}-\overset{-}{w}\right),\;k=0,1,\ldots ,M, \end{array}$$

where $\overset{-}{w}$ is the mean of the series and *M* = *n*/4 is the maximum lag. Obviously, ${\overset{\wedge }{\gamma }}_{-k}={\overset{\wedge }{\gamma }}_{k}$.

Then the autocorrelation function (ACF) at lag *k*, denoted by *ρ*_*k*_, can be estimated according to

(8) $$\begin{array}{c} {\overset{\wedge }{\rho }}_{k}=\frac{{\overset{\wedge }{\gamma }}_{k}}{{\overset{\wedge }{\gamma }}_{0}}. \end{array}$$

Based on the covariance and the resulting ACF, we present the calculation for the PACF at lag *k*, denoted by *α*_*kk*_, as follows:

(9) α∧11=ρ∧1,α∧k+1,k+1=ρ∧k+1−∑j=1kρ∧k+1−jα∧kj1−∑j=1kρ∧jα∧kj,α∧k+1,j=α∧kj−α∧k+1,k+1·α∧k+1,k−j+1,(j=1,2,…,k),

where *k* = 1,2,…, *M*.

In the modeling process of ONN, TNN, and WTT-TNN, the PACF is adopted to find the potential existing relation between the subseries and their lags.

## 3. Evaluation Criteria

To identify the best model quantitatively, three criteria were used to evaluate and compare the models. These criteria included the mean absolute error (MAE), the root mean square error (RMSE), and the mean absolute percentage error (MAPE). MAE, RMSE, and MAPE are measures of the deviation between actual values and forecasting values. The forecasting performance is better when the values of these measures are smaller, and the definitions of these criteria can be found as follows:

(10) $$\begin{array}{c} \text{MAE}=\frac{1}{n}\overset{n}{\underset{t=1}{\sum }}\left|e\left(t\right)\right|,\\ \text{RMSE}={\left(\frac{1}{n}\overset{n}{\underset{t=1}{\sum }}e{\left(t\right)}^{2}\right)}^{1/2},\\ \text{MAPE}=\frac{1}{n}\overset{n}{\underset{t=1}{\sum }}\left|\frac{e\left(t\right)}{y\left(t\right)}\right|, \end{array}$$

where *e*(*t*) = *y*(*t*) − *d*(*t*), *n* is the sample size, and *y*(*t*) and *d*(*t*) are the actual and forecasting values at time period *t*, respectively. Currently, the wind speed forecasted by the MAPE ranges from 25% to 40%. These wind speed predictions depend on the forecasting methods, forecasting horizon, and wind speed characteristics at a given location. In general, the shorter forecasting horizons correspond to more stable wind speed variations and smaller forecasting errors. Otherwise, the forecasting error will increase [49].

## 4. Experimentation Design and Results

### 4.1. Datasets

**Figure 3:**
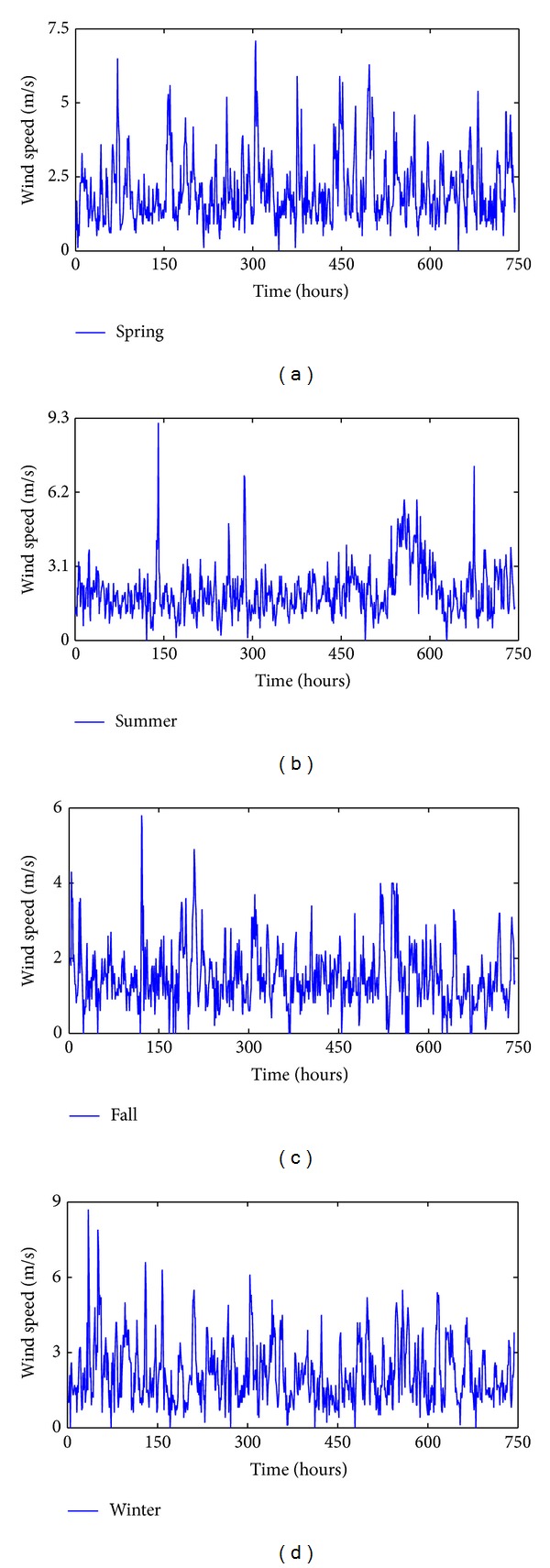
Original wind speed series for the four seasons: (a) spring, (b) summer, (c) fall, and (d) winter.

The hybrid forecasting system presented in this paper has been applied to forecast Hexi Corridor of China's wind speed. The 24 hourly mean wind speed data are collected from January 1, 2010, to April 30, 2011. To reduce the impact of seasonal pattern on wind speed forecasting, the following months are randomly selected: March 2010, July 2010, October 2010, and January 2011, corresponding to the four seasons of the year.  Figure 3 shows an hourly wind speed time series in the four seasons. In the four cases, every case has 744 data. To verify the performance of the proposed hybrid model, the 1–600th ones of this original series are utilized to establish models and the 601–744th ones are utilized to check the validity of the established models.  Table 1 shows the calculation results of the descriptive statistical analysis for the data in Figure 3. In Table 1, it can be observed that the statistical measures of the time series are considerably different among them which are convenient in order to see if the proposed methodology can be applied for different conditions.

### 4.2. Wavelet Decomposition

**Figure 4:**
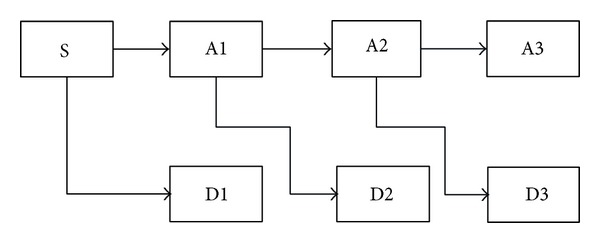
Multilevel decomposition process.

**Figure 5:**
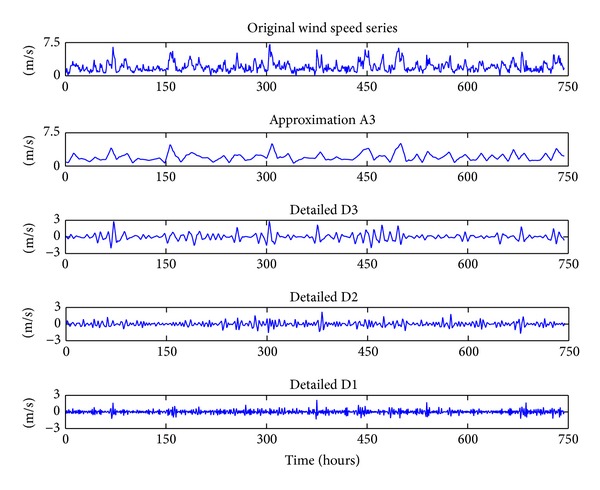
DWT results in spring.

The WTT converts a wind speed series into a set of constitutive series. These constitutive series present a better behavior than the original wind speed series, and therefore they can be predicted more accurately. The reason for the better behavior of the constitutive series is the filtering effect of the WTT. In the WTT literature, a lot of wavelet functions are used for wavelet decomposition. According to the difference of resolution capability and efficiency, a wavelet function of type Daubechies of order 3 (abbreviated as Db3) is used as the mother wavelet in this paper. Also, considering the characteristics of the experimental data, three decomposition levels are considered, since it describes the wind speed series in a more thorough and meaningful way than the others. Three-level decomposition process is shown in Figure 4.  Figure 5 shows the decomposition process of the original wind speed series in spring. From Figure 5, it can be seen that the original wind speed series has been decomposed into a low-pass filter (A3) and three high-pass filters (D1, D2, and D3). The low-pass filter is used to capture the approximated and low frequency nature of the data, whereas the high-pass filter is used to capture the detailed and high-frequency nature of the data. They will be used to build their corresponding TNN forecasting models, respectively. Similarly, the decomposition process of the others can also be got.

### 4.3. Model Structure Determination

#### 4.3.1. Determining the Input Data Order for Forecasting Model

**Figure 6:**
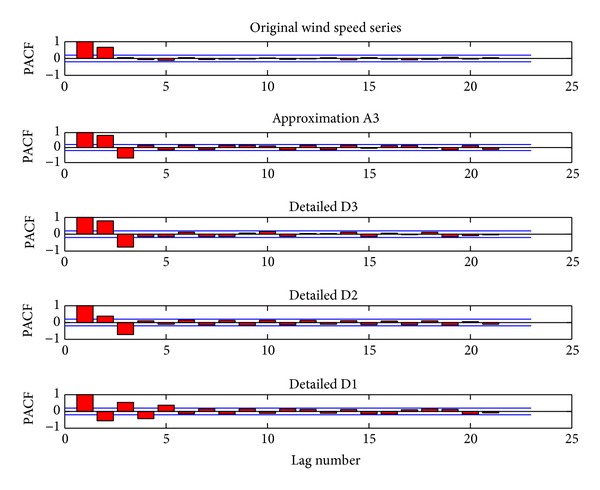
The PACFs of the original wind speed series, low-pass filter, and three high-pass filters (spring).

In order to overcome the limitation of ignoring the relationship between input(s) and output(s) of TNN, inspired from the identification of parameter *p* in ARMA (*p*, *q*) model, the PACF is utilized to identify the inputting data structure of the TNN models.  Figure 6 shows the plots of PACF against the lag length in spring. According to the potential existing relation between the wind subseries and their lags, the input numbers of forecasting models are decided. Similarly, the plots of PACF in others can be shown.  Table 2 lists them.

#### 4.3.2. Determining the Number of Nodes in the Two Hidden Layers

In the modeling process of TNN, it is very important to choose the number of the hidden-layer nodes. Since there are no general rules for choosing them, they are chosen by experimental simulation in our study. On the other hand, according to Kolmogorov's theorem, in the modeling of one-hidden-layer neural network, a hidden layer of 2*n* + 1 nodes is sufficient to map any function for *n* input [50]. Therefore, for model comparison, the total nodes number of two hidden layers is selected as the 2*n* + 1 for *n* input in the modeling process of TNN. In order to further confirm the nodes number in each hidden layer, the experimental simulation is made by using the 1–600th series of all the original wind speed series and subseries. To estimate the performance of each run of the experimental simulation, the MSE is used. Each simulation is run at least 30 times to obtain the mean values. The results of the experimental simulation in spring are shown in Table 3. Similarly, the results of the experimental simulation in other seasons can be got. The optimal network structure of all the original series and subseries is listed in Table 4.

### 4.4. Forecasting Results

**Figure 7:**
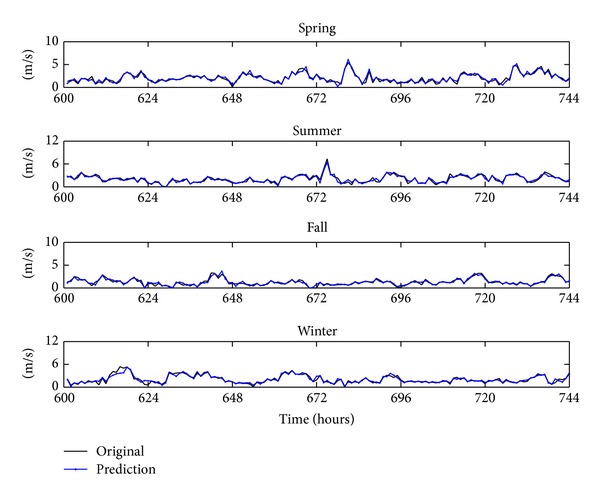
The prediction results of the original wind speed series by the proposed approach.

In the previous section, apply the WTT to decompose an original wind speed series into a set of different subseries, use the TNN to build a forecasting model for each subseries, and make the prediction in each subseries. In this section, the final prediction of the original wind speed data is got by making aggregate calculation for forecasting in subseries.  Figure 7 shows the forecasting results of the four original wind speed series by the proposed approach. In order to validate the forecasting capacity of the proposed hybrid approach, the model comparison is given in the next section.

### 4.5. Model Comparison

The PM, also known as a “Naive Predictor,” is generally used as a benchmark for comparing other tools for short-term wind speed forecasting. Wind speed forecasting methods are usually first tested against the PM in order to evaluate its performance. To evaluate the performance of the proposed approach, in this paper, the WTT-TNN is compared with PM, ONN, and TNN. The comparison results are shown in Table 5 and it can be clearly seen that the proposed approach consistently has the minimum statistical MAE, RMSE, and MAPE. It is concluded that the proposed approach can improve the forecasting performance and is an effective approach.

### 4.6. Significance Test

In order to test whether the proposed WTT-TNN model is superior to the PM, ONN, and TNN in wind speed forecasting, the Wilcoxon signed-rank test is adopted. The test is a nonparametric statistical hypothesis test that does not require any normal distribution assumption in the data and deals with the signs and ranks of the values and not with their magnitude. It is one of the most commonly adopted tests in evaluating the predictive capabilities of two different models to see whether there is statistically significant difference between them [51–55].

The test procedure first calculates the differences between the paired observations, ranks them from the smallest to the largest by absolute value, and then affixes the sign of each difference to the corresponding rank [53, 54]. The sum of the ranks having a plus sign is called *J*+, and the sum of the ranks having a minus sign is called *J*−. When the sample size *n* is larger than 25, the distribution of *J* (where either *J*+ or *J*− may be used for *J*) is closely approximated by a normal distribution with a mean of *u*_*J*_ = *n*(*n* + 1)/4 and a standard error of $\sigma_{J}=\sqrt{n\left(n+1\right)(2n+1)/24}$ [53]. Thus the test statistic can be calculated from *Z* = (|*J* − *u*_*J*_| − 0.5)/*σ*_*J*_, where for *J* we may use, with identical results, either *J*+ or *J*−. For the details of the Wilcoxon signed-rank test, please refer to Diebold and Mariano [51] and Pollock et al. [54].

We used this test to evaluate the predictive performances of the four models.  Table 6 contains the resulting* z*-statistic values and *P* values from the two-tailed Wilcoxon signed-rank test comparing between the proposed WTT-TNN and the other three models, and the numbers in parentheses denote the corresponding *P* values. In this study, the significance level is *α* = 0.05 and *z*_critical_ = 1.96.  Table 6 shows that each* z*-statistic value is greater than 1.96 and each *P* value is less than 0.05. Therefore, we decide that the proposed WTT-TNN model was significantly different from the other three models. Because the proposed method can be used to generate the smallest error in the four datasets, we concluded that this method is significantly better for forecasting wind speed relative to the other three models.

## 5. Conclusions

The accurate wind speed forecasting can be very useful for wind parks management and wind power utilization. To this purpose, a novel hybrid approach known as WTT-TNN is proposed for wind speed forecasting. A WTT is used to decompose wind speed into an approximate scale and several detailed scales. The approximated scale reveals the trend, while the detailed scales tend to be related to seasonal influences and exogenous variables effect. Then, a TNN is used to predict both approximated scale and detailed scales, respectively. In order to find the optimal network architecture, the PACF is adopted to determine the number of neurons in the input layer, and an experimental simulation is made to determine the number of neurons within each hidden layer in the modeling process of TNN. Afterwards, the final prediction value can be obtained by the sum of these prediction results. To evaluate the performance of the proposed approach, it is applied to forecast Hexi Corridor of China's wind speed. Compared with the PM, the ONN, and the TNN, simulation results in four different cases show that the proposed method increases wind speed forecasting accuracy.

## Figures and Tables

**Table 1:** Descriptive statistics of seasonal wind speed data.

	Mean (m/s)	Std. dev. (m/s)	Maximum (m/s)	Minimum (m/s)
Spring	2.03	1.11	7.10	0.00
Summer	2.05	1.05	9.10	0.00
Fall	1.50	0.82	5.80	0.00
Winter	2.15	1.24	8.70	0.00

**Table 2:** Input numbers of neural network models for wind speed series.

Case	Series				
	Original	A3	D3	D2	D1
Spring	2	3	3	3	5
Summer	2	3	4	3	5
Fall	2	3	3	3	6
Winter	2	3	3	3	6

**Table 3:** The choice of nodes number of the hidden layers in spring.

Case	Series	The total nodes number in two hidden layers	The nodes number in the first hidden layer	The nodes number in the second hidden layer	Error (MSE)
Spring	Original	5	1	4	0.0134
			2	3	0.0135
			**3**	**2**	**0.0133**
			4	1	0.0136
	A3	7	1	6	0.0015
			2	5	0.0017
			3	4	0.0018
			4	3	0.0016
			5	2	0.0015
			**6**	**1**	**0.0014**
	D3	7	1	6	0.0024
			2	5	0.0015
			3	4	0.0017
			4	3	0.0014
			**5**	**2**	**0.0012**
			6	1	0.0016
	D2	7	1	6	0.0043
			**2**	**5**	**0.0022**
			3	4	0.0054
			4	3	0.0034
			5	2	0.0037
			6	1	0.0041
	D1	11	1	10	0.0040
			2	9	0.0037
			3	8	0.0039
			**4**	**7**	**0.0032**
			5	6	0.0036
			6	5	0.0038
			7	4	0.0034
			8	3	0.0036
			9	2	0.0048
			10	1	0.0035

**Table 4:** The optimal network structure of all the original series and subseries.

Case	Series	The nodes number in the input layer	The nodes number in the first hidden layer	The nodes number in the second hidden layer	The nodes number in the output layer
Spring	Original	2	3	2	1
	A3	3	6	1	1
	D3	3	5	2	1
	D2	3	2	5	1
	D1	5	4	7	1

Summer	Original	2	4	1	1
	A3	3	4	3	1
	D3	4	2	7	1
	D2	3	5	2	1
	D1	5	4	7	1

Fall	Original	2	3	2	1
	A3	3	3	4	1
	D3	3	4	3	1
	D2	3	4	3	1
	D1	6	8	5	1

Winter	Original	2	4	1	1
	A3	3	4	3	1
	D3	3	3	4	1
	D2	3	4	3	1
	D1	6	7	6	1

**Table 5:** The prediction results using PM, ONN, TNN, and WTT-TNN.

Case	Errors	PM	ONN	TNN	WTT-TNN
Spring	MAE (m/s)	0.7021	0.6384	0.5866	0.2659
	RMSE	0.9178	0.8520	0.8019	0.3317
	MAPE	0.4961	0.4748	0.3949	0.1999

Summer	MAE (m/s)	0.6535	0.6124	0.6040	0.2595
	RMSE	0.8903	0.8218	0.8106	0.3345
	MAPE	0.4872	0.4653	0.3855	0.1891

Fall	MAE (m/s)	0.7015	0.6302	0.4767	0.2312
	RMSE	0.6942	0.6745	0.6514	0.4540
	MAPE	0.3535	0.3267	0.2820	0.1769

Winter	MAE	0.7076	0.6275	0.5918	0.2702
	RMSE	0.8101	0.7666	0.7398	0.3967
	MAPE	0.4436	0.4083	0.3566	0.1949

**Table 6:** Wilcoxon signed-rank test comparing between WTT-TNN and PM, ONN, and TNN.

Case	Models	PM	ONN	TNN
Spring	WTT-TNN	6.72 (0.00)	5.41 (0.00)	3.48 (0.00)
Summer		5.98 (0.00)	4.67 (0.00)	3.29 (0.00)
Fall		5.37 (0.00)	4.85 (0.00)	3.02 (0.00)
Winter		6.59 (0.00)	5.33 (0.00)	3.14 (0.00)

The numbers in parentheses are the corresponding *P* values.
